# A case report of a mammary gland type adenocarcinoma of the vulva in a patient with a concomitant breast cancer: a diagnostic challenge

**DOI:** 10.3389/fonc.2026.1716250

**Published:** 2026-01-20

**Authors:** Ilenia Nobile, Antonia Tessitore, Mario Palumbo, Dominga Boccia, Chiara Mignogna, Giuseppe Bifulco, Luigi Della Corte

**Affiliations:** 1 Department of Public Health, School of Medicine, University of Naples “Federico II”, Naples, Italy; 2 Department of Translational Medical Sciences, School of Medicine, University of Naples “Federico II”, Naples, Italy; 3 Department of Neuroscience, Reproductive Sciences and Dentistry, School of Medicine, University of Naples “Federico II”, Naples, Italy

**Keywords:** anogenital mammary-like glands, case report, mammary-like adenocarcinoma, synchronous breast cancer, vulvar carcinoma

## Abstract

We describe the case of a 68‐year‐old woman who presented with a 4‐cm ulcerated lesion in the left paraclitoral area. Imaging revealed increased FDG uptake at the vulvar lesion and multiple skeletal sites but no suspicious inguinal lymphadenopathy, while breast imaging showed a 30‐mm BI‐RADS 5 lesion in the upper outer quadrant of the left breast. Core biopsies confirmed two distinct primaries: an invasive ductal carcinoma of the breast and an eccrine ductal‐type adenocarcinoma of the vulva arising from anogenital mammary‐like glands. A bone biopsy demonstrated metastatic breast carcinoma. The patient underwent radical anterior vulvectomy with bilateral sentinel lymph node biopsy; final pathology confirmed a well‐differentiated, adenocarcinoma of mammary‐gland type of the vulva (pT1bN0) with negative margins. Because of breast metastases, systemic therapy with ribociclib and letrozole was initiated but later discontinued owing to therapy‐related acute myeloid leukemia. At 9‐month follow‐up, no recurrence of the vulvar disease was observed. This report highlights one of the very few documented instances of synchronous mammary‐like vulvar carcinoma and breast carcinoma. It underscores the diagnostic complexity of the case and emphasizes the importance of an individualized, multidisciplinary approach tailored to tumor biology, staging, and patient comorbidities.

## Introduction

The occurrence of mammary-like tissue in the vulvar region is extremely rare and derives from the presence of vestigial structures known as anogenital mammary-like glands (AGMLGs) (1). These ectopic glands are thought to originate from the embryonic mammary ridge, which typically regresses except in the thoracic region but may persist in ectopic locations along the “milk line,” including the vulva. First described histologically in the 19th century and further characterized by van der Putte in the late 20th century, AGMLGs exhibit striking morphological and functional similarities to normal breast tissue (2). They respond to hormonal stimuli and can undergo a full range of benign and malignant transformations, such as fibrocystic changes, fibroadenomas, intraductal papillomas, and adenocarcinomas (3).

Although primary malignancies arising from AGMLGs are exceptionally uncommon, when they do occur, their histopathological and immunophenotypic resemblance to orthotopic breast carcinoma creates significant diagnostic challenges. In particular, the distinction between a primary mammary-like carcinoma of the vulva and a metastasis from breast cancer can be exceedingly difficult and requires an integrated assessment of histopathological, immunohistochemical, and clinical data (4).

To date, only a few cases of synchronous mammary-like carcinoma of the vulva and breast carcinoma have been reported in the literature. This overlap further complicates the diagnostic pathway, as the possibility of metastatic disease must be carefully weighed against the rare occurrence of two independent but phenotypically similar primaries (4, 5).

Herein, we describe an exceptionally rare case of adenocarcinoma of mammary gland type (AMGT) of the vulva, diagnosed in a woman with concomitant breast cancer and FDG-avid skeletal lesions. Initially suspected to be vulvar metastasis from breast carcinoma, the vulvar lesion was ultimately confirmed to be a distinct primary tumor arising from AGMLGs. This case underscores the importance of a multidisciplinary approach and highlights the diagnostic complexity and therapeutic implications of such rare clinical scenarios.

## Case report

A 68-year-old white woman, G2P2, referred to our Azienda Ospedaliera Universitaria Federico II of Naples for evaluation of a newly symptomatic vulvar lesion. Her medical history was remarkable for hypertension, type II diabetes mellitus and hypercholesterolemia. There was no family history of cancers. Gynecological examination revealed a hard, non-tender, ulcerated heteroplastic lesion approximately 4 cm in diameter in the left paraclitoral area. No inguinal lymphadenopathy was appreciable. Vulvoscopy confirmed a lesion superficially confined to the midline but extended deeper behind it, without involvement of the urethra (**Supplementary Figure S1**). Multiple biopsies were obtained. While awaiting histological results, further diagnostic investigations were prescribed, including abdominal and pelvis magnetic resonance imaging (MRI) with and without contrast, tumor markers (carcinoembryonic antigen, CA-125, CA 15.3, CA 19.9, alpha-fetoprotein, SCC antigen), 18F-FDG positron emission tomography (PET), and total-body computed tomography (CT) with and without contrast. Abdomen and pelvis MRI heteroplastic thickening of the vulvar walls with heterogeneous contrast enhancement (18 mm in thickness and 40 mm in anteroposterior extension), without significant abdominal or pelvic lymphadenopathy, but with bilateral inguinal lymph node micronodularity. Among the tumor markers tested, only CA 15.3 was elevated (46.2 U/ml versus a cutoff of 32.5 U/ml). The 18F-FDG PET scan showed thickening of vulvar tissue in the left paramedian region with increased glucose uptake (SUV max 6.2), as well as a mild but relevant uptake by an irregular parenchymal density in the upper outer quadrant of the left breast (SUV max 3.7). There were also multiple skeletal lesions with focal FDG uptake, especially in the spine, ribs, pelvis, and left scapula. The total-body CT with and without intravenous contrast and multiplanar reconstruction (MPR) confirmed asymmetric parenchymal density (maximum diameter approximately 24 mm) in the left breast, in addition to vulvar wall thickening.

This raised a complex issue in the differential diagnosis between a primary breast carcinoma with vulvar metastasis versus primary vulvar lesion occurring concomitantly with breast carcinoma.

**Figure 1:**
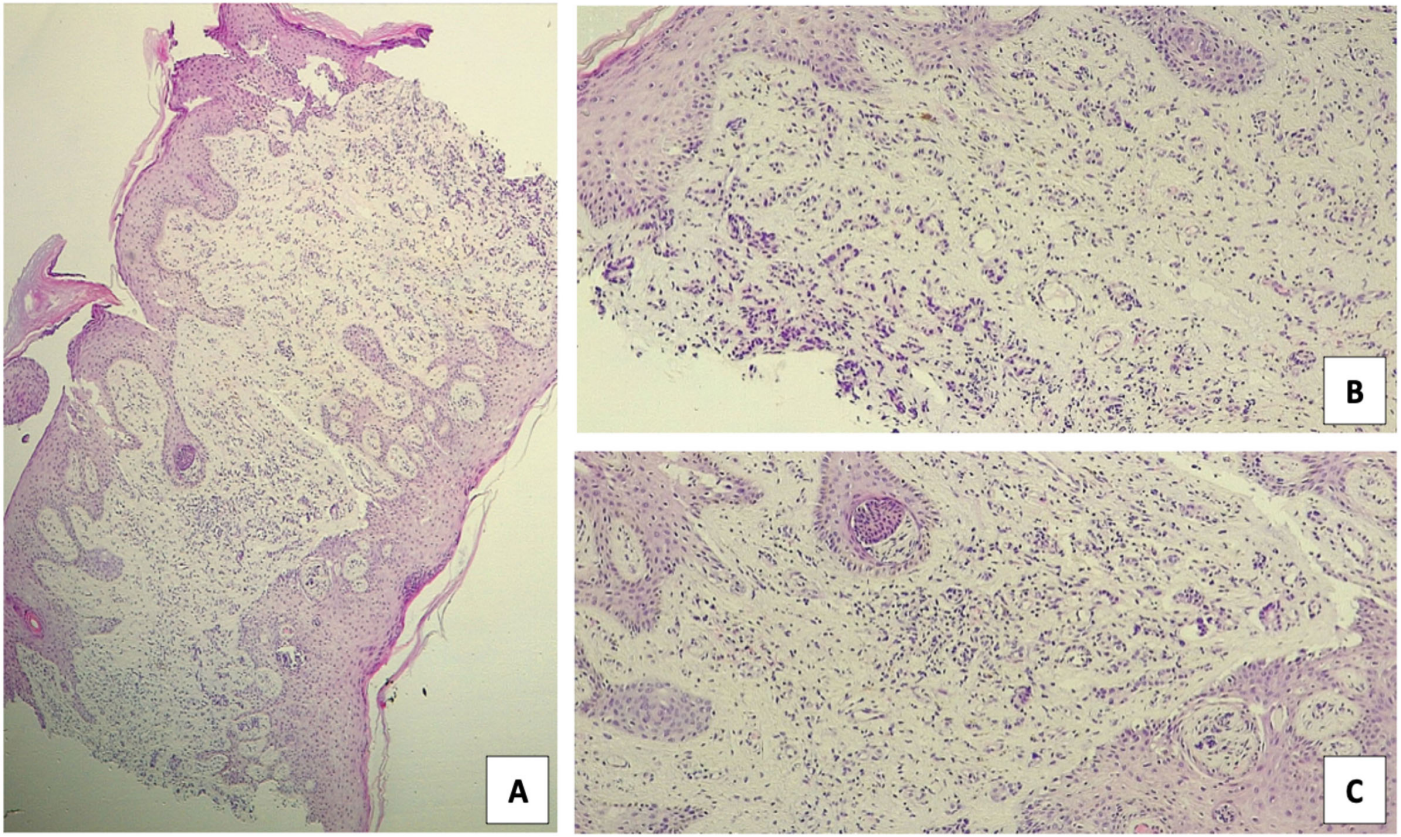
Histological examination of the vulvar biopsy **(A)** Low-power overview (40x): epithelial thickening with architectural disarray and invasive neoplastic proliferation extending into the stroma, and **(B, C)** Intermediate magnification (100X): irregular glandular proliferation with infiltrative growth pattern and associated desmoplastic stromal reaction, consistent with adenocarcinoma morphology.

**Figure 2:**
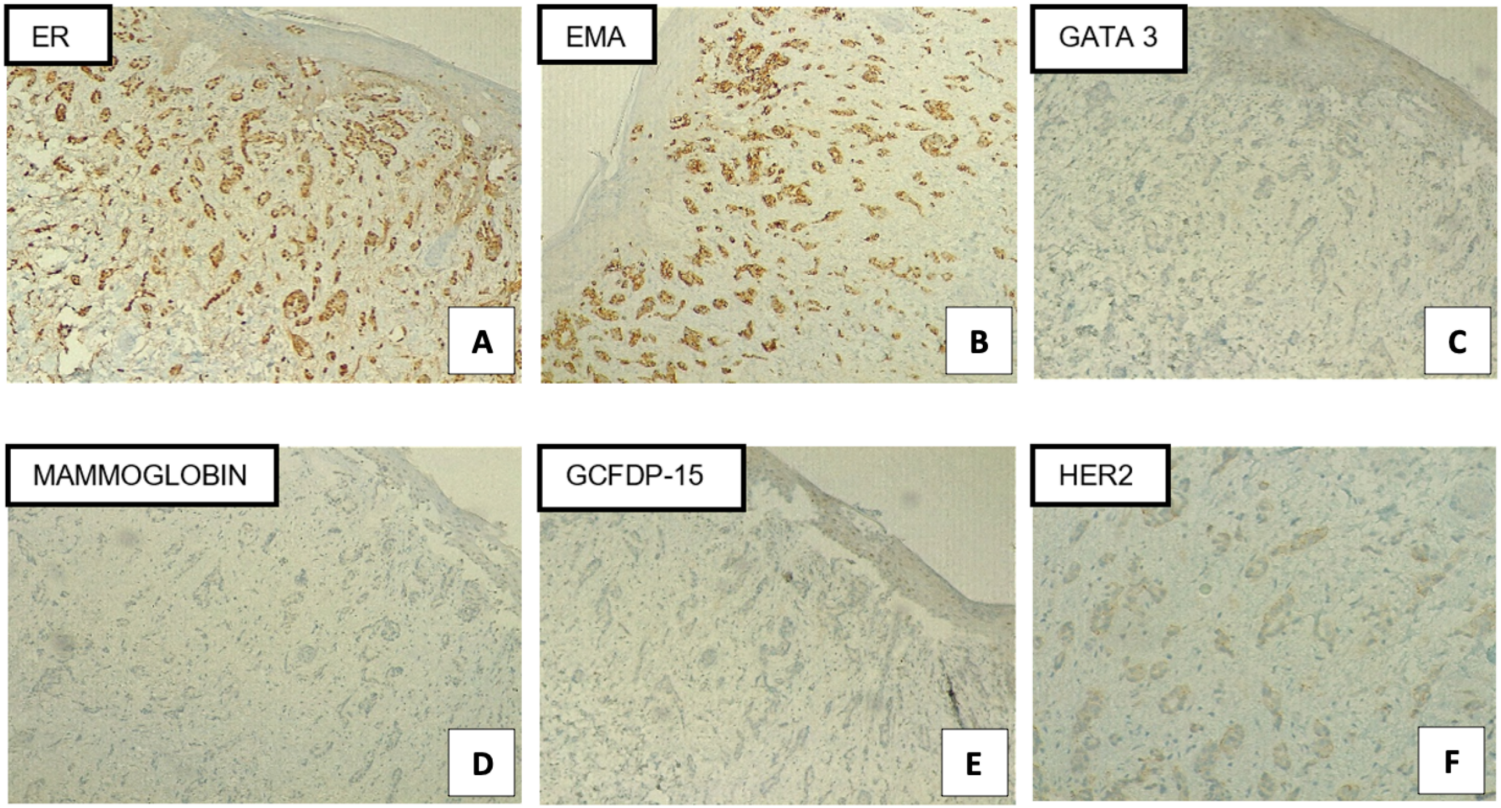
Immunohistochemical profile of vulvar biopsy (100X): **(A)** ER (Estrogen Receptor): strong nuclear positivity in neoplastic cells, **(B)** EMA (Epithelial Membrane Antigen): diffuse cytoplasmic and membranous positivity, **(C)** GATA-3: negative or very faint staining not supportive of urothelial/breast lineage, **(D)** Mammaglobin: negative, **(E)** GCDFP-15: negative, **(F)** HER2: incomplete faint membrane staining and within > 10% of invasive tumor cells (score 1+).

Bilateral contrast-enhanced mammography revealed a spiculated nodular opacity with heterogeneous density and microcalcifications in the upper outer quadrant of the left breast (maximum diameter 30 mm), resulting in architectural distortion. This lesion was classified as BI-RADS 5 (**Supplementary Figure S2**). Ultrasound-guided core needle biopsy of the left breast was performed. Histological examination revealed invasive breast carcinoma of moderate grade (E-cadherin+, GATA3+, p63−), with over 50% neoplastic cellularity. Immunohistochemical analysis demonstrated ER+ in 90% of tumor cells, PR+ in 80%, Ki67 expression in approximately 20%, and HER2 (C-erB2) score 2+ (complete moderate membranous staining in more than 10% of tumor cells). The lesion was categorized as B5b according to the 2024 GIPAM classification. FISH analysis for HER2 gene amplification was negative. In order to investigate the skeletal lesions in PET, a bone biopsy was performed. It confirmed metastases from breast carcinoma. Initial histopathologic evaluation of the vulvar biopsy suggested an eccrine ductal-type carcinoma. However, the final surgical specimen shows infiltration by a well-differentiated adenocarcinoma, with a predominantly glandular and partly tubular pattern, within a fibrotic stroma. In light of the immunohistochemical profile and the presence of *in situ* neoplasia, the case is interpreted primarily as a primary vulvar lesion with features of mammary gland-type adenocarcinoma (according to WHO 5th edition). (**Figures 1**, **2**).

**Figure 3:**
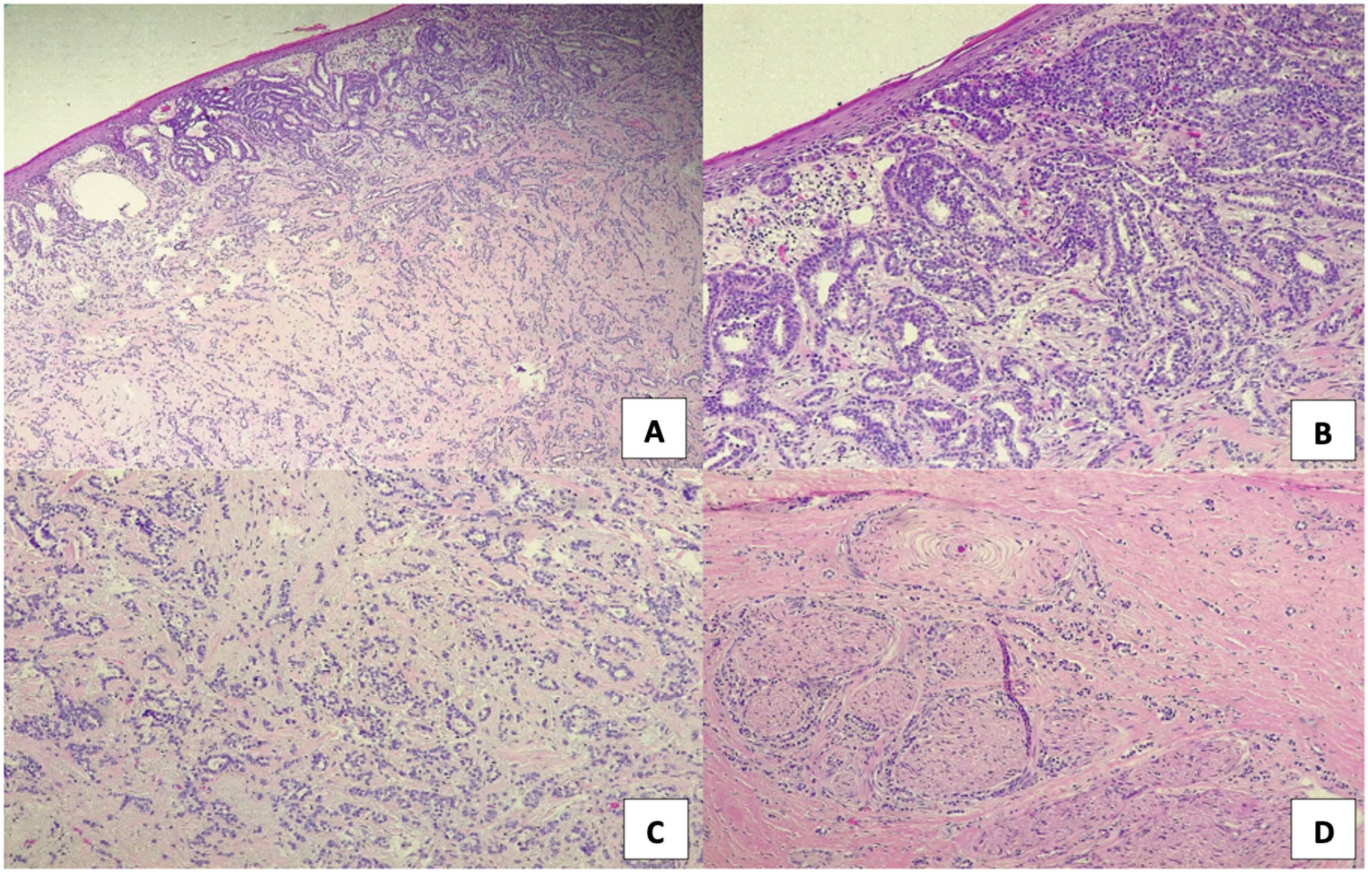
Key morphological features of vulvar resection **(A)** Low-power (40X magnification) panoramic view of the neoplastic proliferation, **(B)** Area showing carcinoma *in situ* associated with the invasive component (100x magnification), **(C)** A detail of glandular morphology of the neoplasm, infiltrating derma (200x magnification), and **(D)** Widespread perineural invasion, indicative of aggressive biological behavior (200x magnification).

The patient was initiated on treatment with letrozole in combination with CDK inhibitors. The case was discussed in our tumor board discussion and, after a long discussion including a scientific literature review, the surgical intervention was recommended. The patient underwent radical anterior vulvectomy with bilateral inguinal sentinel lymph node biopsy, after first performing inguinal lymphoscintigraphy the day before surgery (**Supplementary Figure S3**). To minimize the risk of postoperative complications in a patient with substantial comorbidities, sentinel nodes were identified bilaterally at Daseler level IV and were excised through a 3cm skin incision. A radical anterior vulvectomy was performed down to the deep fascia, with an additional 2mm deep resection down to the pubic bone, and 1cm lateral margins on both sides. As outlined above, the final histology of the vulvar lesion confirmed the diagnostic hypothesis. The immunohistochemical profile was consistent with mammary-like adenocarcinoma of the vulva: EMA+, ER+, GATA3 negative or very faint staining, negative for GCDFP-15 and Mammaglobin (**Supplementary Figure S4**). Extensive perineural invasion was observed. The tumor was 4 mm from the deep surgical margin and clear for 1 cm from all other margins. Sentinel lymph nodes were negative for malignancy. The final pathological staging was pT1b N0, according to AJCC 8th edition (**Figure 3** and **Supplementary Figure S4**). Currently, after the first vulvar follow-up at 9 months, the patient is free from disease relapse but has developed, for reasons that are still unclear, acute myeloid leukemia with NPM1 mutation which was promptly treated. She underwent a 18F-FDG PET scan, which did not show tracer uptake in the examined body regions, except for mild uptake at the osteomedullary level (**Supplementary Figure S5**). The patient’s clinical timeline is summarized in **Supplementary Table S1**.

## Discussion

This article presents a rare case of mammary-like adenocarcinoma arising within ectopic vulvar mammary tissue, concomitant with primary breast carcinoma. This malignant neoplasm typically affects multiparous women aged 60 years or older. The most frequent site of occurrence is the labia major and usually it presents as solitary nodules. The most common subtype is adenocarcinoma identical to invasive ductal carcinoma of the breast; other types, for example lobular, ductal-lobular, or tubulolobular variants, have occasionally been reported. All of these subtypes show histopathological features like their mammary counterparts, including E-cadherin positivity in ductal subtypes (6, 7). Rarely, mutations in genes of the PI3K/AKT pathway have been identified. Prognostically, anogenital mammary-like glands adenocarcinomas appear locally aggressive and are associated with lymph node metastases in approximately 60% of cases, although variant-specific clinical outcomes remain poorly defined due to their rarity (8–10).

In the literature, the first plausible case of a mammary-like vulvar carcinoma synchronous with breast cancer dates back to 1976. Guerry et al. (5) described a patient with a history of right breast carcinoma who subsequently developed contralateral breast cancer and a vulvar lesion, consisted of ectopic mammary tissue with both intraductal and invasive carcinoma. For the first time, this case raised the complex question of the differential diagnosis between a primary mammary-like vulvar carcinoma synchronous with breast cancer and a vulvar metastasis of mammary origin. We identified only another case report of vulvar mammary-like carcinoma concomitant with breast carcinoma, published in 2006. Intra et al. (6), in their study published in the *International Journal of Gynecological Cancer*, provide a comprehensive description of the patient’s management which comprised left hemivulvectomy, bilateral inguinal sentinel lymph node biopsy, and radioguided resection of the breast lesion (6). More data are available about isolated cases of mammary-like carcinoma of the vulva. Between 1936 and 2022, a total of 41 cases of AMGT has been reported (7). Most of these tumors expressed estrogen and progesterone receptors. The management of mammary-like carcinomas of the vulva represents one of the most complex challenges in gynecologic pathology (11, 12). The rarity of these tumors, combined with their morphological, immunohistochemical, and molecular similarity to breast carcinomas, makes both diagnosis and therapeutic planning extremely difficult (13). Primarily, diagnostic assessment must include differential diagnosis with the main vulvar malignancies such as vulvar squamous cell carcinoma, extramammary Paget’s disease (14–16), and primary vulvar adenocarcinoma. The main clinical, histological, immunohistochemical, and therapeutic differences are summarized in **Supplementary Table S2**.

The main diagnostic challenge is to distinguish between a primary mammary-like carcinoma of the vulva from a cutaneous metastasis of breast carcinoma, or from the coexistence of two distinct but phenotypically similar primary neoplasms (16). Currently, the criteria proposed for the diagnosis of mammary-like carcinoma of the vulva are: morphological similarity to breast carcinoma, positive estrogen and/or progesterone receptor expression, positivity for typical immunohistochemical breast markers, and the presence of carcinoma *in situ* or non-neoplastic mammary-type tissue adjacent to the tumor, after excluding primary breast carcinoma or metastases from other organs (17–19). It is even more difficult in cases with a clinical history of breast cancer or when an AMGT is associated with synchronous or metachronous breast carcinoma, as in our patient. Immunohistochemical profiles, which are often overlapping, are not always definitive for differentiation so the integration of clinical data, imaging, and through histopathological evaluation becomes essential to guide diagnosis (20, 21). Therapeutic management also raises important questions. There are no standardized protocols for the treatment of mammary-like carcinomas of the vulva and clinical management is generally based on the approach used for breast cancer (22, 23). The main therapeutic strategies employed in these cases are reported in the literature since 2013 (**Table 1**) (24, 25). The predominant therapeutic approach involved surgery combined with sentinel lymph node biopsy or lymphadenectomy (25). In some cases, surgical management was supplemented with adjuvant therapy. Bogani et al. (17) additionally reported the use of neoadjuvant therapy. In other studies, patient management relied exclusively on systemic medical treatment (21, 23, 25).

**Table 1:** Summary of main reported cases of mammary-like glands of the vulva since 2013.

Study and country	Age at diagnosis (years)	Immunohistochemical analysis	Surgical treatment	Medical treatment	Outcome
Bogani G et al. (2013), Italy. (17)	71	ER+, PR+, HER2-, CK7+, GATA3+, CK20-	Radical vulvectomy	Neoadjuvant CHT + Adjuvant antiestrogen hormone therapy	24 months DF
Butler et al. (2013), USA. (18)	65	ER+, PR-, HER2-	Wide local excision + SNLB	Aromatase inhibitor	18 months DF
Benito V et al. (2013), Spain. (19)	82	ER+, PR+, HER2-, GATA3+, EMA+, CK7+, CK20-	Wide local excision + IFLD	Adjuvant hormone therapy	18 months DF
Baykal et al. (2014), Turkey. (20)	73	ER+, PR+, HER2-	Radical vulvectomy + IFLD	RT + CHT	NA
Grewal et al. (2017), Canada. (21)	60	ER-, HER2+, CK7+, CK20-, GATA3+	NP	CHT + target anti-HER therapy	No clinical response
Ishigaki T et al. (2017), Japan. (22)	72	ER+, PR+, HER2-, GATA3+	Local excision with positive margins, followed by wide local excision + SNLB	Adjuvant hormone therapy	6 months DF
Al-Mansouri et al. (2018), Australia. (23)	76	ER+, PR+, HER2-	NP	RT + Aromatase inhibitor	NA
Niakan et al. (2020), USA. (24)	58	ER-, PR-, HER2+, GATA3+, EMA+, CK7+, CK20-	Wide local excision + SNLB	RT + adjuvant target anti-HER therapy	18 months DF
Song Q et al. (2024), Cina. (25)	68	ER+, PR+, CK7+, GATA3+, EMA+	NP	CHT + RT	NA

SLNB, sentinel lymph node biopsy; IFLD, inguinofemoral lymphadenectomy; NP, not performed; NA, not applicable, RT, radiotheraphy; CHT, chemotherapy; DF, disease free.

The coexistence of a mammary-like carcinoma of the vulva with a primary breast neoplasm raises several important clinical considerations. One of the key questions is whether these lesions should be managed as two independent diseases or rather as manifestations of a single neoplastic process. Equally challenging is the decision on systemic therapy—whether it should be shared across both sites or tailored to the biology of each tumor. Another crucial aspect is determining which of the two malignancies will have the greatest influence on overall prognosis and follow-up strategy. In cases involving aggressive biological subtypes, such as HER2-positive or triple-negative tumors, treatment may require the use of targeted agents or intensive chemotherapy. Although these regimens are not routinely validated for vulvar carcinomas, they may still be justified by the molecular behavior of the neoplasm. Overall, the concurrence of a mammary-like carcinoma of the vulva and breast carcinoma represents a highly complex diagnostic and therapeutic scenario, underscoring the absence of standardized treatment guidelines. It demands a specialized, multidisciplinary, and individualized approach, as exemplified by the present case.

This case report presents several limitations that may affect the generalizability of its findings. The extreme rarity of mammary-like adenocarcinoma of the vulva inherently restricts the ability to draw firm conclusions regarding diagnostic criteria or optimal treatment strategies, while the coexistence of metastatic breast cancer represents a significant confounding factor that may have influenced both imaging interpretation and clinical decision-making. Moreover, the absence of comparative molecular analysis between the two neoplasms, beyond immunohistochemistry, limits the possibility of definitively excluding a clonal relationship. As with all single-patient descriptions, the clinical trajectory and therapeutic response observed here may not be representative of the broader population.

Despite these limitations, the case offers important clinical insights. The striking morphological and immunophenotypic similarity between mammary-like carcinoma of the vulva and primary breast carcinoma underscores the value of an integrative diagnostic workflow that combines histopathology, radiology, and clinical evaluation. Furthermore, the presence of synchronous breast cancer highlights the risk of misinterpreting a primary vulvar tumor as metastatic disease, reinforcing the importance of targeted biopsy and accurate clinicopathologic correlation. The absence of standardized treatment protocols for mammary-like vulvar carcinomas also emphasizes the need for clinicians to individualize management strategies, often adapting principles from breast oncology, while carefully balancing surgical morbidity and patient comorbidities. The case additionally illustrates the crucial role of a multidisciplinary tumor board in ensuring diagnostic precision and in tailoring treatment approaches in scenarios where therapeutic decisions for one malignancy may influence the clinical course of the other.

Future research should focus on improving diagnostic accuracy and therapeutic decision-making in patients with multiple synchronous tumors involving the breast and vulvar region. Comparative molecular profiling, such as next-generation sequencing, methylation studies, or clonality assessment, may contribute to distinguishing true dual primaries from metastatic disease with greater certainty. The development of standardized diagnostic algorithms for lesions arising from anogenital mammary-like glands, including structured immunohistochemical panels, could further reduce the risk of misclassification. Finally, the establishment of international registries and multicenter collaborations would facilitate the accumulation of larger datasets, enabling the formulation of shared treatment recommendations and follow-up strategies. Advances in hybrid imaging modalities and radiomics may also enhance early detection and characterization of vulvar and breast lesions with overlapping morphologic or metabolic features, thereby improving clinical outcomes in similar complex scenarios.

## Conclusion

Synchronous adenocarcinoma of mammary gland type (AMGT) of the vulva and primary breast carcinoma is an extremely rare and diagnostically challenging condition. This case emphasizes the importance of distinguishing between vulvar metastasis and dual primaries, a process that requires an integrated approach involving clinical history, imaging, histopathology, and immunohistochemistry. The absence of standardized treatment guidelines necessitates highly individualized management, taking into account tumor biology, disease stage, comorbidities, and patient preferences. Our report highlights the role of multidisciplinary tumor boards in guiding appropriate surgical and systemic interventions and underlines the need for further collaborative research to establish evidence-based recommendations for this uncommon clinical scenario.

## Patient perspective

The patient reported initial anxiety and confusion regarding the presence of two distinct cancers diagnosed almost simultaneously. She expressed relief in receiving clear explanations from the medical team and appreciated the coordinated approach that combined gynecologic oncology, breast oncology, radiology, and pathology. Despite the burden of undergoing surgery and systemic therapy, she felt reassured by the close follow-up and valued being involved in the decision-making process. At her latest follow-up, she emphasized the importance of timely diagnosis and comprehensive counseling for patients facing rare and complex conditions like hers.

## Data Availability

The raw data supporting the conclusions of this article will be made available by the authors, without undue reservation.

## Supplementary material

Supplementary Figure 1 Vulvoscopy confirmed the lesion.

Supplementary Figure 2 Spiculated nodular opacity with heterogeneous density and microcalcifications in the upper outer quadrant of the left breast (Laterality: L, Viewposition: CC, KVP: 34kV, Tube current: 200mA, Scale: 0.18).

Supplementary Figure 3 Vulvar lesion in left paraclitoral area and after surgery with catheter.

Supplementary Figure 4 Additional immunohistochemical panel of vulvar resection **(A)** ER: strong and diffuse nuclear staining, **(B)** Mammaglobin: negative, and **(C)** GCDFP-15: negative.

Supplementary Figure 5 18F-FDG PET scan during follow up (KVP: 120kV, Tube current:10mA, Location: 0mm, Thickness: 1671.82mm, Spacing:10mm, Scale: 0.14).
