# Supplementary figures and images for: A case report of a mammary gland type adenocarcinoma of the vulva in a patient with a concomitant breast cancer: a diagnostic challenge
Source: Front Oncol. 2026 Jan 20;16:1716250. doi: 10.3389/fonc.2026.1716250 (PMC12864080; doi:10.3389/fonc.2026.1716250)

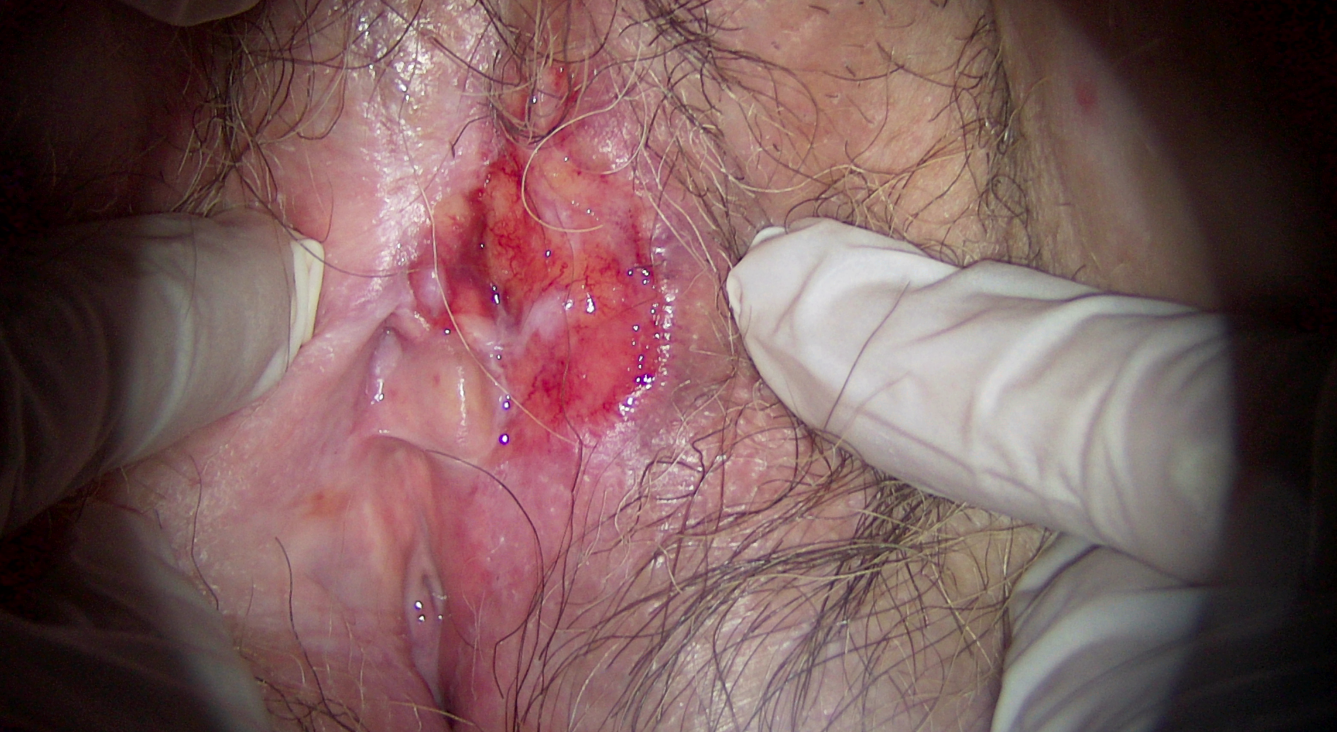

Supplement: Supplementary Figure 1 — Vulvoscopy confirmed the lesion. [file Image1.png]

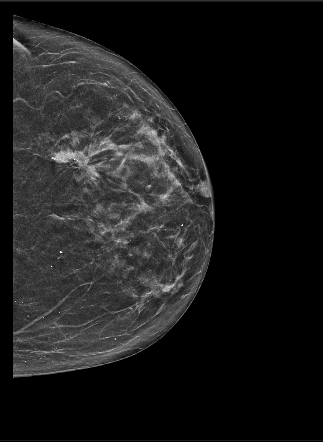

Supplement: Supplementary Figure 2 — Spiculated nodular opacity with heterogeneous density and microcalcifications in the upper outer quadrant of the left breast (Laterality: L, Viewposition: CC, KVP: 34kV, Tube current: 200mA, Scale: 0.18). [file Image2.png]

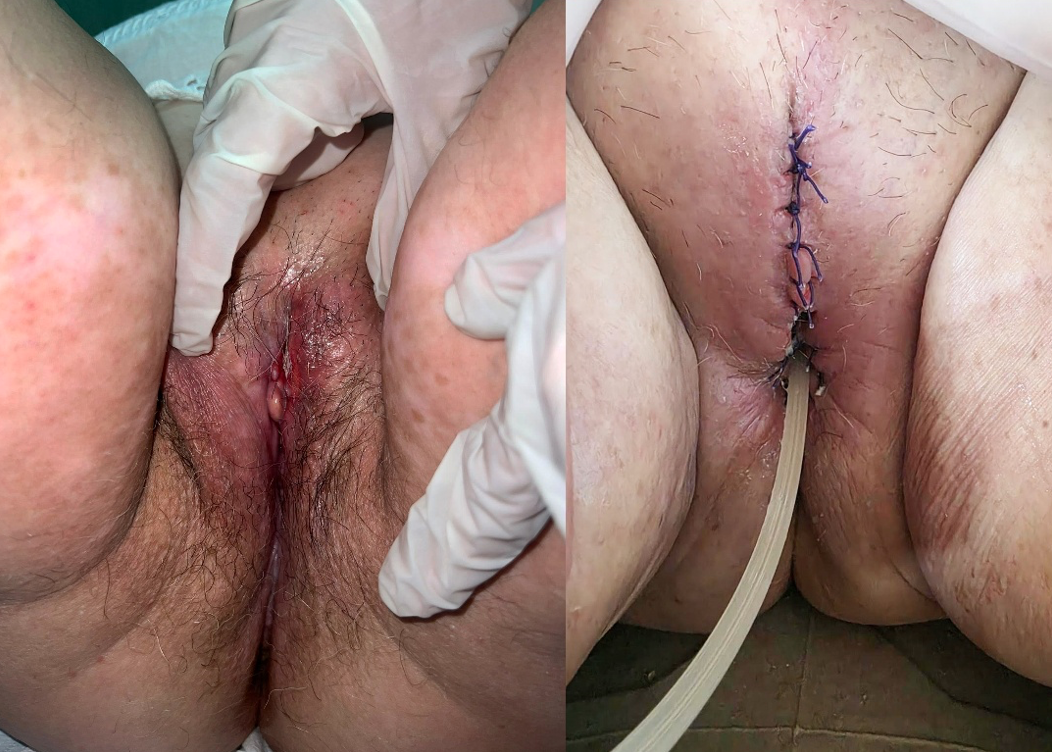

Supplement: Supplementary Figure 3 — Vulvar lesion in left paraclitoral area and after surgery with catheter. [file Image3.png]

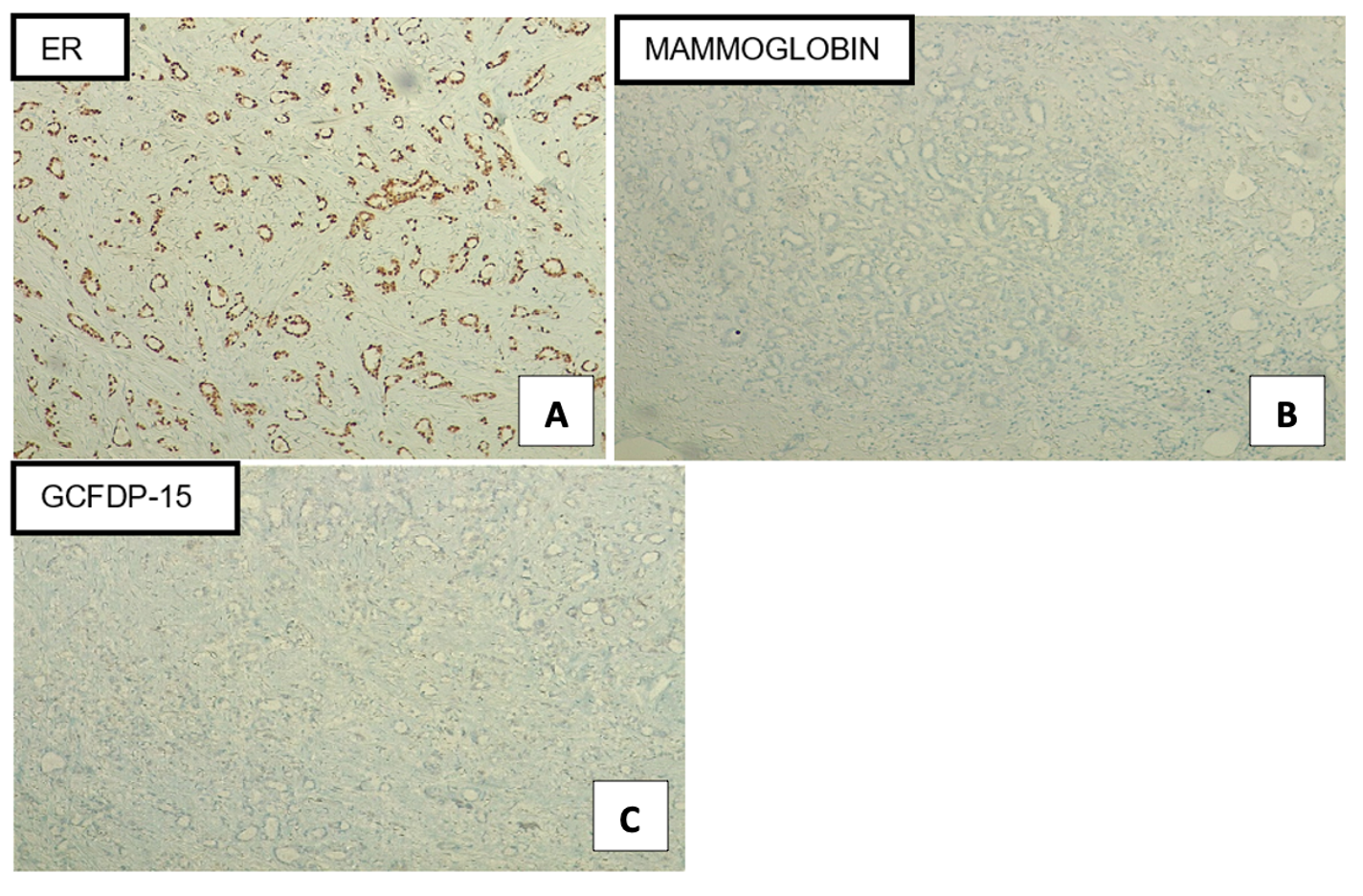

Supplement: Supplementary Figure 4 — Additional immunohistochemical panel of vulvar resection (A) ER: strong and diffuse nuclear staining, (B) Mammaglobin: negative, and (C) GCDFP-15: negative. [file Image4.png]

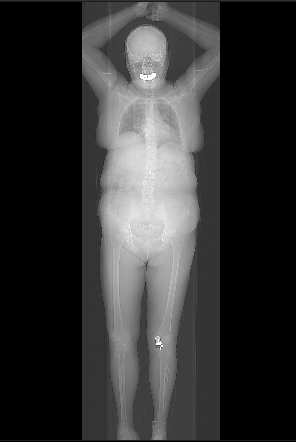

Supplement: Supplementary Figure 5 — 18F-FDG PET scan during follow up (KVP: 120kV, Tube current:10mA, Location: 0mm, Thickness: 1671.82mm, Spacing:10mm, Scale: 0.14). [file Image5.png]
