# Supplementary material for: A case report of a mammary gland type adenocarcinoma of the vulva in a patient with a concomitant breast cancer: a diagnostic challenge
Source: Front Oncol. 2026 Jan 20;16:1716250. doi: 10.3389/fonc.2026.1716250 (PMC12864080; doi:10.3389/fonc.2026.1716250)
Supplement: Supplementary file 6 [file Supplementaryfile1.docx]

**Table S1.** Timeline of the clinical course, interventions, and outcomes.

| **Time point** | **Event/Findings** |
| --- | --- |
| **Presentation (Month 0)** | 68-year-old woman with a 4 cm ulcerated paraclitoral vulvar lesion. |
| **Initial imaging** | PET/CT: FDG-avid vulvar lesion and multiple skeletal foci; no inguinal nodes. |
| **Concurrent breast findings** | Mammography/US: 30 mm BI-RADS 5 lesion, upper outer quadrant, left breast. |
| **Biopsies** | Vulva: adenocarcinoma of mammary gland type (ER+, EMA+). Breast: invasive ductal carcinoma (ER+/PR+/HER2−). Bone biopsy: metastatic breast carcinoma. |
| **Surgical treatment** | Radical anterior vulvectomy with bilateral sentinel lymph node biopsy. |
| **Systemic therapy** | Ribociclib + letrozole started (for metastatic breast carcinoma). |
| **Therapy modification** | Discontinuation due to therapy-induced acute myeloid leukemia. |
| **Follow-up**  **(9 months)** | No evidence of vulvar recurrence. Ongoing surveillance for breast carcinoma. |

**Table S2:** Differential Diagnosis of Major Vulvar Lesions.

| Lesion | Clinical features | Histology | Immunohistochemistry | Treatments |
| --- | --- | --- | --- | --- |
| Mammary-like carcinoma of the vulva | Vulvar nodule, often on the labia majora | Morphology similar to breast carcinoma | ER+, PR+, GATA3+, mammaglobin+/-; CK7+; CK20- | Surgery (local excision or vulvectomy ± lymph nodes biopsy/ lymphadenectomy), ± adjuvant therapy as for breast cancer |
| Vulvar squamous cell carcinoma | Ulcerated, painful lesion, often associated with itching | Proliferation of atypical squamous cells, variable keratinization | p63+, CK5/6+, p16+ (HPV+); ER/PR- | Surgery (local excision or vulvectomy ± lymph nodes biopsy/ lymphadenectomy), ± chemo/radiotherapy |
| Extramammary Paget's disease | Chronic erythematous, pruritic, eczematous lesions | Intraepithelial Paget cells with pale/mucinous cytoplasm | CK7+, CEA+, variable HER2; ER/PR- | Wide local excision ± radiotherapy |
| Primary vulvar adenocarcinoma | Rare vulvar nodule | Glandular adenocarcinoma not related to mammary-like glands | Variable, profile; generally CK7+, ER/PR− | Surgery (local excision or vulvectomy ± lymph nodes biopsy/ lymphadenectomy); ± chemo/radiotherapy |
